# Regional differences in self-reported HIV care and management in the EuroSIDA study

**DOI:** 10.7448/IAS.17.4.19504

**Published:** 2014-11-02

**Authors:** Kamilla Grønborg Laut, Amanda Mocroft, Jeffrey Lazarus, Peter Reiss, Jürgen Rockstroh, Igor Karpov, Aza Rakhmanova, Brygida Knysz, Santiago Moreno, Panagiotis Gargalianos, Jens Lundgren, Ole Kirk, on behalf of Eurosida in Eurocoord

**Affiliations:** 1 Department of Infectious Diseases, Rigshospitalet, University of Copenhagen, Copenhagen, Denmark; 2 Department of Infection and Population Health, University College London, London, UK; 3 Academic Medical Center, University of Amsterdam and Stichting HIV Monitoring, Amsterdam, Netherlands; 4 Immunologische Ambulanz, University Hospital Bonn, Bonn, Germany; 5 Department of Infectious Diseases, Belarus State Medical University, Minsk, Belarus; 6 Department of Infectious Diseases, Botkin Hospital St Petersburg, Russian Federation; 7 Department of Infectious Diseases, Wroclaw University School of Medicine, Wroclaw, Poland; 8 Servicio Enfermedades Infecciosas, Hospital Ramon y Cajal, Madrid, Spain; 9 Infectious Diseases Unit, 1st Internal Medicine Department, G Gennimatas Hospital, Athens, Greece

## Abstract

**Introduction:**

EuroSIDA has previously reported a poorer clinical prognosis for HIV-positive individuals in Eastern Europe (EE) as compared with patients from other parts of Europe, not solely explained by differences in patient characteristics. We explored regional variability in self-reported HIV management at individual EuroSIDA clinics, with a goal of identifying opportunities to reduce the apparent inequalities in health.

**Methods:**

A survey (www.chip.dk/eurosida/csurvey) on HIV management was conducted in early 2014 in all currently active EuroSIDA clinics. Responders in EE were compared with clinics in all other EuroSIDA regions combined (non-EE). Characteristics were compared between regions using Fishers exact test.

**Results:**

A total of 80/97 clinics responded (82.5%, 12/15 in EE, 68/82 in non-EE). Participating clinics reported seeing a total of 133,532 patients [a median of 1300 per clinic (IQR 700–2399)]. The majority of clinics requested viral load and CD4 measurements at least every six months for patients on as well as off ART (EE 66.7%, non-EE 75%, *p*=0,72). Significantly fewer EE clinics performed resistance tests before ART as well as upon treatment failure (Figure 1). Half of the EE clinics indicated following WHO guidelines (EE 50%, non-EE 7.4%, *p*<0.0001), whereas most non-EE clinics followed EACS guidelines (non-EE 76.5%, EE 41.7%, *p*=0.017). The majority of EE clinics and ¼ non-EE clinics indicated deferral of ART initiation in asymptomatic individuals until CD4 ≤350 cells/mm^3^ (Figure 1). There were no significant regional differences in screening haematology, liver or renal function, which the majority of clinics reported to do routinely. However, EE clinics reported screening significantly less for cardiovascular disease (CVD), and only about half screened for tobacco use, alcohol consumption and drug use (Figure 1). Screening for cervical cancer and for anorectal cancer was low in both regions (Figure 1).

**Conclusions:**

We found significant regional variability in self-reported HIV management across Europe, with less resistance testing, screening for CVD and substance use in EE. EE clinics indicated deferral of ART initiation for longer than non-EE clinics. Adherence to international guidelines for cervical cancer screening was poor in both regions. Whether differences in HIV management are reflected in clinical outcomes deserves further investigation.

**Figure 1:**
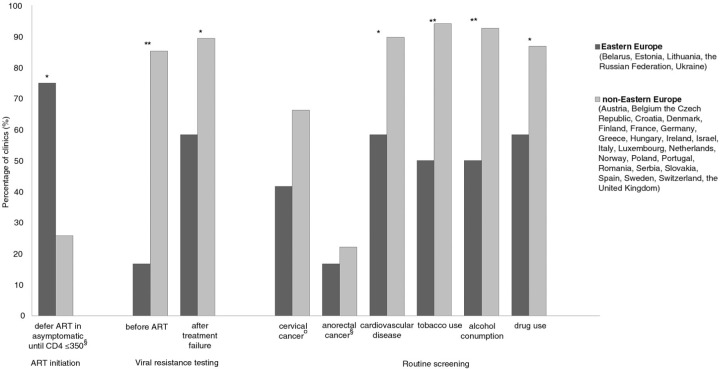
Regional differences in self-reported HIV management: initiation of ART in asymptomatic individuals, viral resistance testing, and routine screening for selected comorbidities. Screening for cervical cancer included performing cervical smear and gynaecological exam. Screening for anorectal cancer included performing anal pap and anorectal exam. § Based on responses from 67/68 non-Eastern European and 12/12 Eastern European clinic. § Based on responses from 67/68 non-Eastern European and 12/12 Eastern European clinic. *<0.05. **p<0.001.

